# Supplementary material for: Thermal optimized PCR coupled to CRISPR-Cas12a for rapid detection of blaOXA-1 resistance gene
Source: PLoS One. 2026 May 15;21(5):e0337675. doi: 10.1371/journal.pone.0337675 (PMC13178884; doi:10.1371/journal.pone.0337675)
Supplement: S1_raw_images [file pone.0337675.s003.pdf]

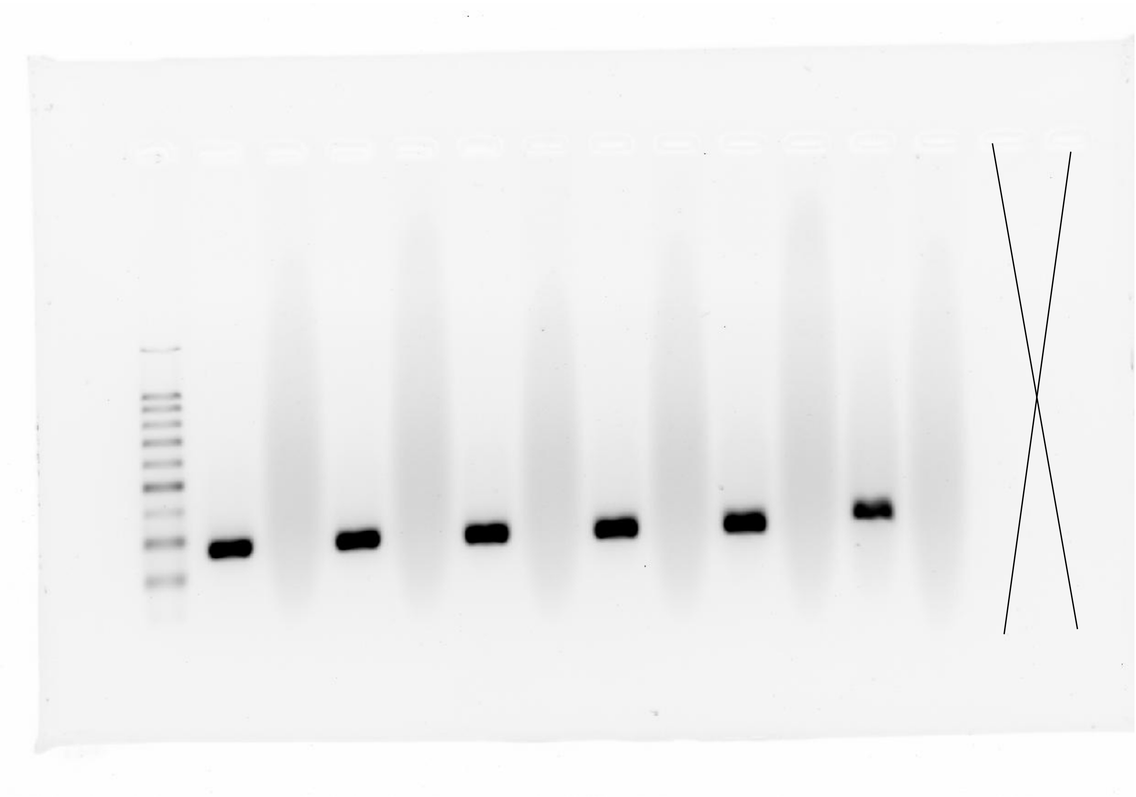

Agarose gel corresponding to Fig. 2A. The image was captured using the GelDoc Go Imaging System (Bio-Rad, USA) with the Blue Tray and default settings for SYBR Gold.

Gel lanes from left to right as follows:

- GeneRuler 100 bp DNA ladder (Cat. N° SM0241, Thermo, USA)
- positive control amplified by standard PCR protocol
- negative control amplified by standard PCR protocol
- positive control amplified by modified PCR protocol with TRR of 0.6 °C/s
- negative control amplified by modified PCR protocol with TRR of 0.6 °C/s
- positive control amplified by modified PCR protocol with TRR of 0.8 °C/s
- negative control amplified by modified PCR protocol with TRR of 0.8 °C/s
- positive control amplified by modified PCR protocol with TRR of 1.2 °C/s
- negative control amplified by modified PCR protocol with TRR of 1.2 °C/s
- positive control amplified by modified PCR protocol with TRR of 1.6 °C/s
- negative control amplified by modified PCR protocol with TRR of 1.6 °C/s
- positive control amplified by modified PCR protocol with TRR of 2.2 °C/s
- negative control amplified by modified PCR protocol with TRR of 2.2 °C/s

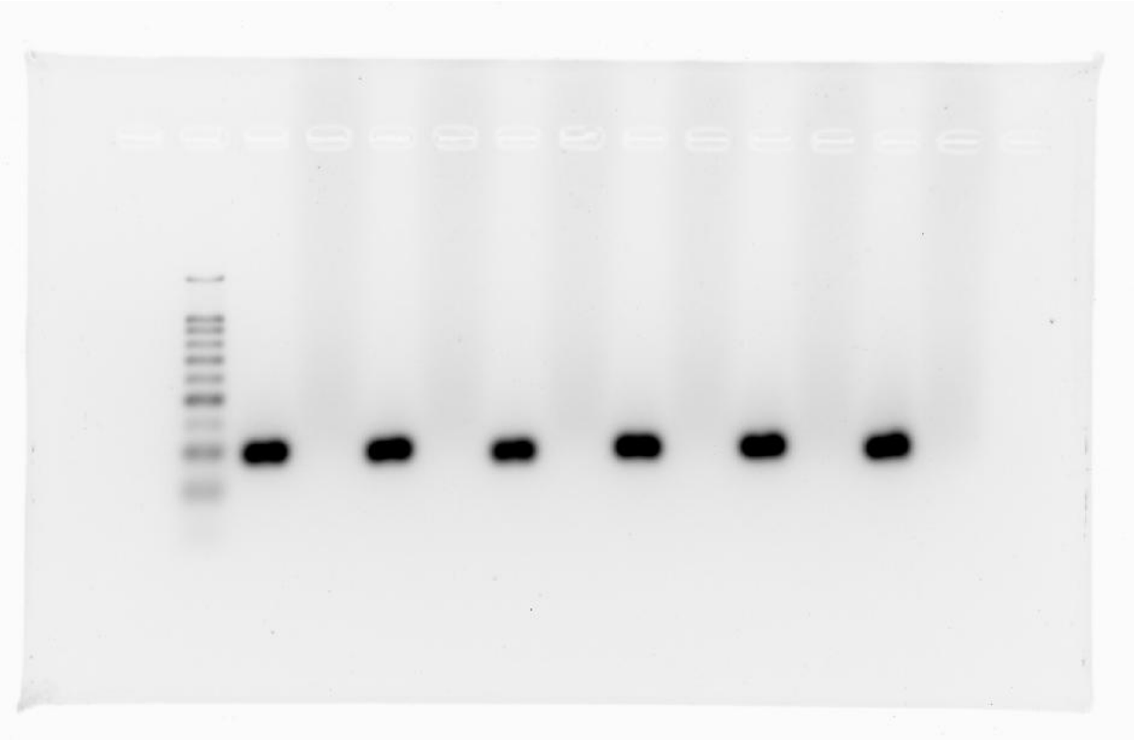

Agarose gel corresponding to Fig. 2B. The image was captured using the GelDoc Go Imaging System (Bio-Rad, USA) with the Blue Tray and default settings for SYBR Gold.

Gel lanes from left to right as follows:

- GeneRuler 100 bp DNA ladder (Cat. N° SM0241, Thermo, USA)
- positive control amplified by standard PCR protocol
- negative control amplified by standard PCR protocol
- positive control amplified by modified PCR protocol with TRR of 0.6 °C/s
- negative control amplified by modified PCR protocol with TRR of 0.6 °C/s
- positive control amplified by modified PCR protocol with TRR of 0.8 °C/s
- negative control amplified by modified PCR protocol with TRR of 0.8 °C/s
- positive control amplified by modified PCR protocol with TRR of 1.2 °C/s
- negative control amplified by modified PCR protocol with TRR of 1.2 °C/s
- positive control amplified by modified PCR protocol with TRR of 1.6 °C/s
- negative control amplified by modified PCR protocol with TRR of 1.6 °C/s
- positive control amplified by modified PCR protocol with TRR of 2.2 °C/s
- negative control amplified by modified PCR protocol with TRR of 2.2 °C/s

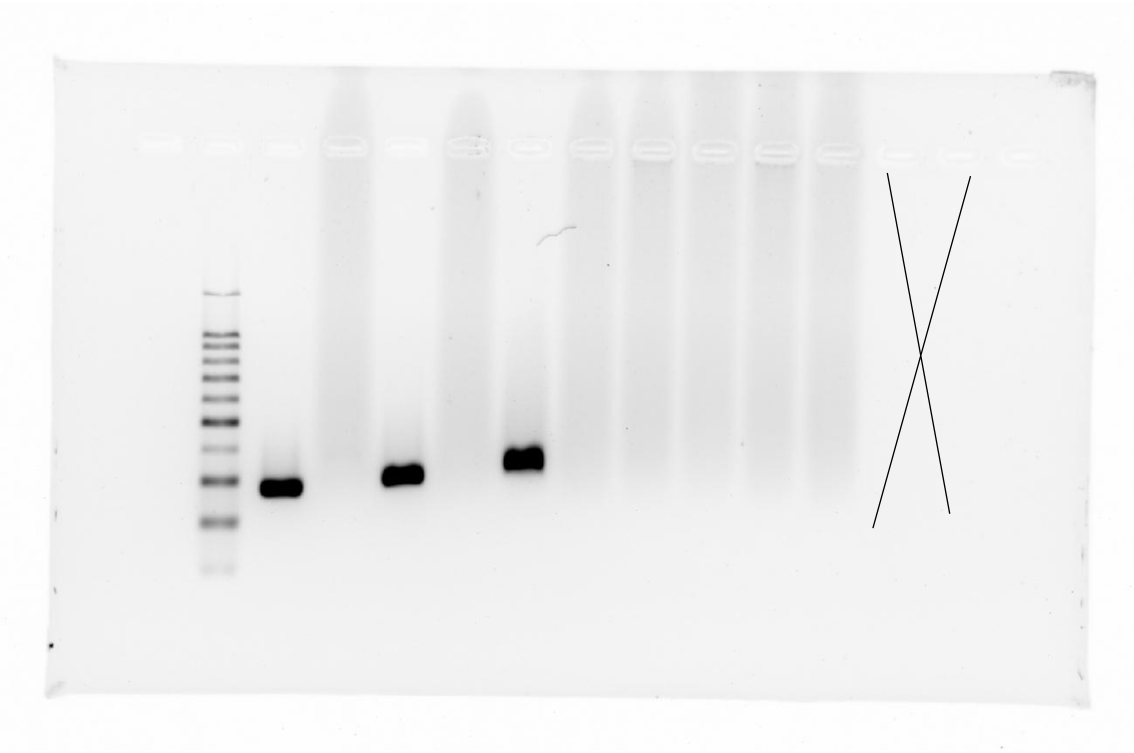

Agarose gel corresponding to S2 Fig. The image was captured using the GelDoc Go Imaging System (Bio-Rad, USA) with the Blue Tray and default settings for SYBR Gold.

Gel lanes from left to right as follows:

- GeneRuler 100 bp DNA ladder (Cat. N° SM0241, Thermo, USA)
- positive control amplified by standard PCR protocol with 30 seg per cycling step
- negative control amplified by standard PCR protocol with 30 seg per cycling step
- positive control amplified by standard PCR protocol with 15 seg per cycling step
- negative control amplified by standard PCR protocol with 15 seg per cycling step
- positive control amplified by standard PCR protocol with 10 seg per cycling step
- negative control amplified by standard PCR protocol with 10 seg per cycling step
- positive control amplified by standard PCR protocol with 5 seg per cycling step
- negative control amplified by standard PCR protocol with 5 seg per cycling step
  positive control amplified by standard PCR protocol with 1 seg per cycling step
- negative control amplified by standard PCR protocol with 1 seg per cycling step

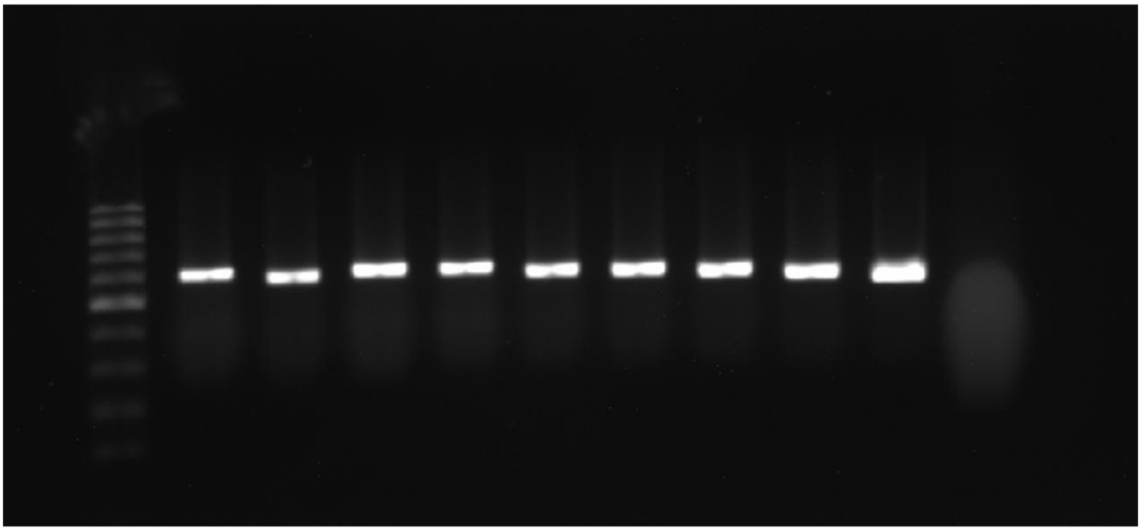

Agarose gel corresponding to S2 Fig. The image was captured using the GelDoc Go Imaging System (Bio-Rad, USA) with the Blue Tray and default settings for SYBR Gold.

Gel lanes from left to right as follows:

- GeneRuler 100 bp DNA ladder (Cat. N° SM0241, Thermo, USA)
- positive control amplified by modified PCR protocol with TRR of 2.2 °C/s
- positive control amplified by modified PCR protocol with TRR of 2.0 °C/s
- positive control amplified by modified PCR protocol with TRR of 1.8 °C/s
- positive control amplified by modified PCR protocol with TRR of 1.6 °C/s
- positive control amplified by modified PCR protocol with TRR of 1.4 °C/s
- positive control amplified by modified PCR protocol with TRR of 1.2 °C/s
- positive control amplified by modified PCR protocol with TRR of 1.0 °C/s
- positive control amplified by modified PCR protocol with TRR of 0.8 °C/s
- positive control amplified by standard PCR protocol
- negative control amplified by standard PCR protocol
