# Supplementary material for: Thermal optimized PCR coupled to CRISPR-Cas12a for rapid detection of blaOXA-1 resistance gene
Source: PLoS One. 2026 May 15;21(5):e0337675. doi: 10.1371/journal.pone.0337675 (PMC13178884; doi:10.1371/journal.pone.0337675)
Supplement: S1 Supporting Information [file pone.0337675.s004.docx]

**Supplementary figures**


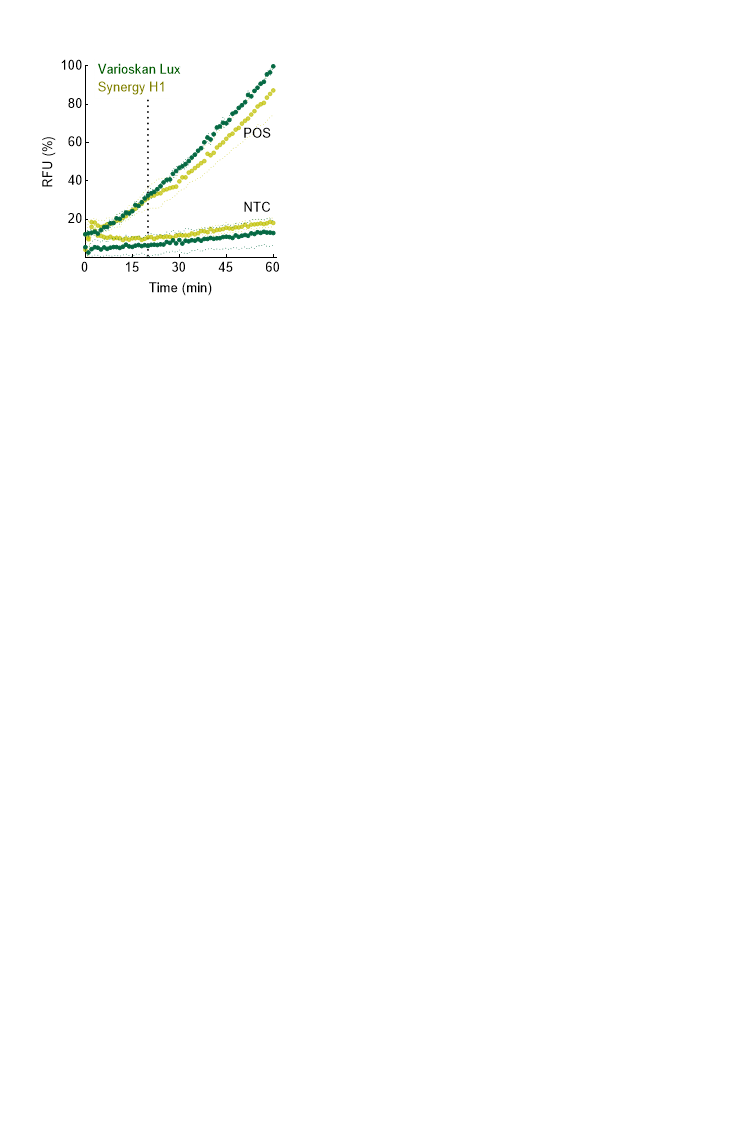


**S1 Fig. Signal readout comparison between two microplate reader instruments.** Positive (POS) and non-template control (NTC) reactions were measured simultaneously in the Synergy H1 and the Varioskan LUX equipment. Fluorescence settings were set to 491 nm excitation and 525 nm emission. Reactions were measured for 60 minutes. Raw fluorescence is plotted as percentage-normalized values for a better comparison. The dotted line on the X-axis represents the time cutoff at 20 minutes. Each reaction was performed in triplicate.


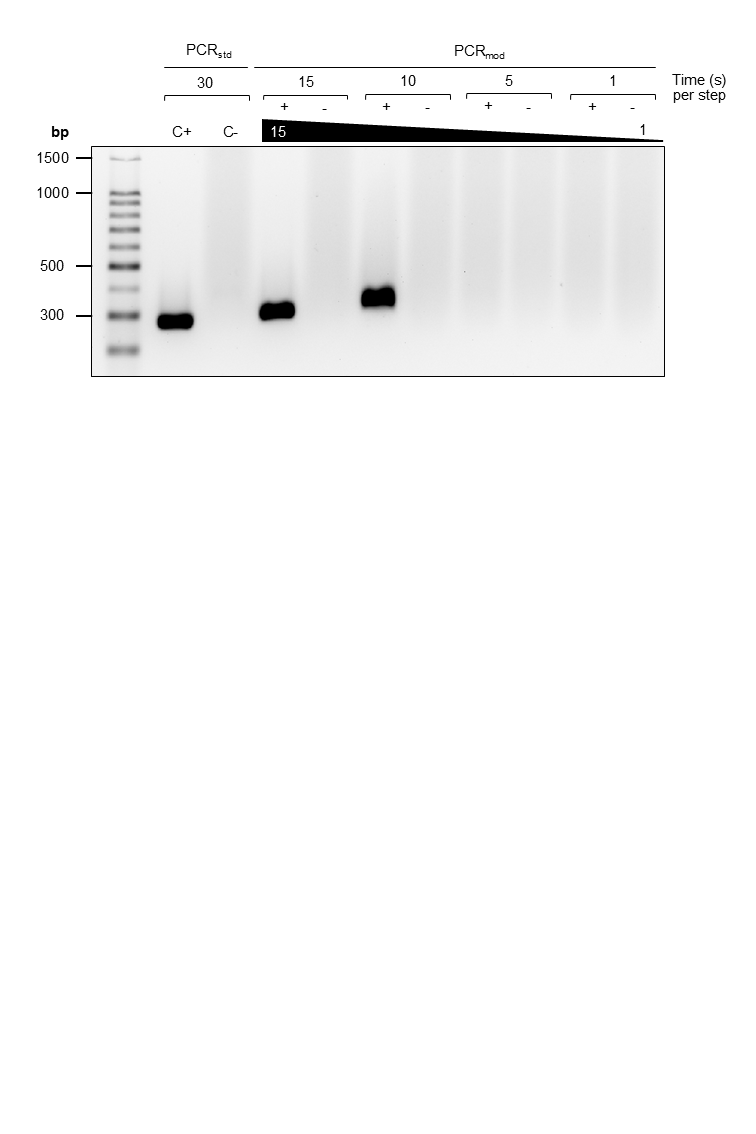


**S2 Fig. Holding-time curve for amplification in the modified PCR cycling using an in-house DNA Taq polymerase.** Amplified 282-bp products obtained from a modified PCR (PCR_mod_) were analyzed using 1.7% agarose gel electrophoresis, with a 100 bp ladder for size reference. Standard PCR (PCR_std_) was used as a positive amplification reaction (C+). The holding-time shown above the gel corresponds to the holding times for the denaturation and annealing steps.


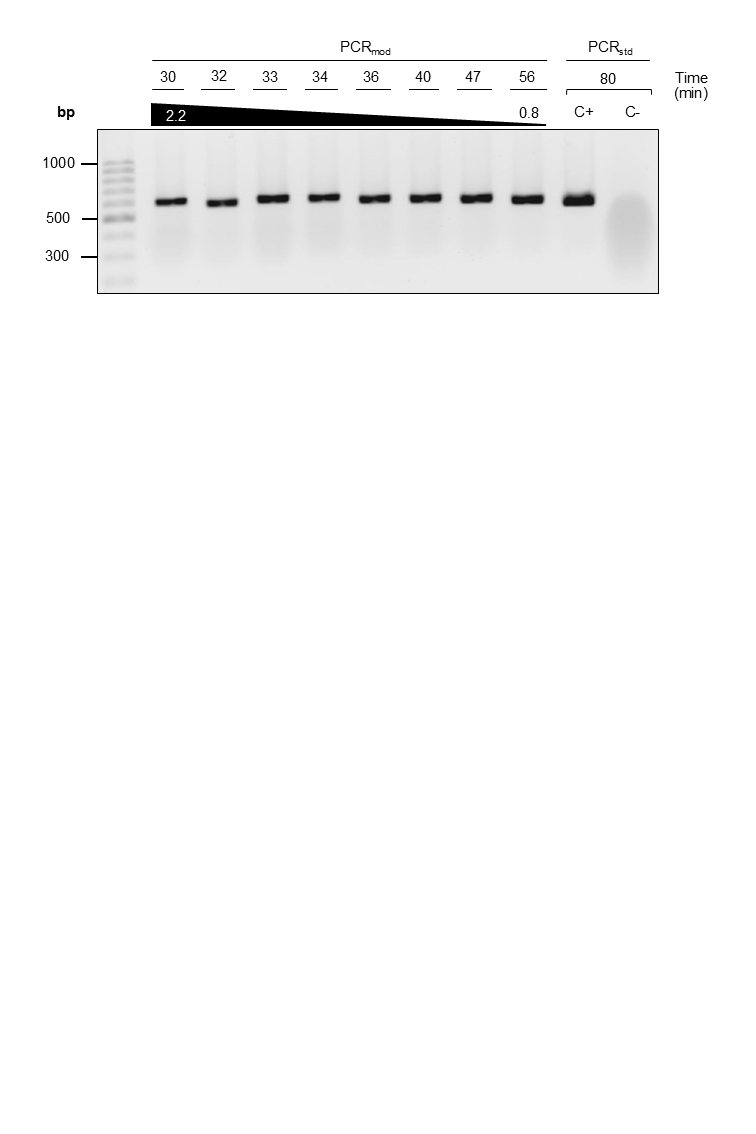


**S3 Fig. Thermal ramp rate (TRR) curve for amplification of 564 bp-amplicon of the *bla_OXA-1_* gene using a commercial DNA Taq polymerase.** Specific amplification was observed for the longer amplicon regardless of the thermal ramp rate evaluated. Amplified products were visualized using 1.7% agarose gel electrophoresis, with a 100 bp ladder for size reference.


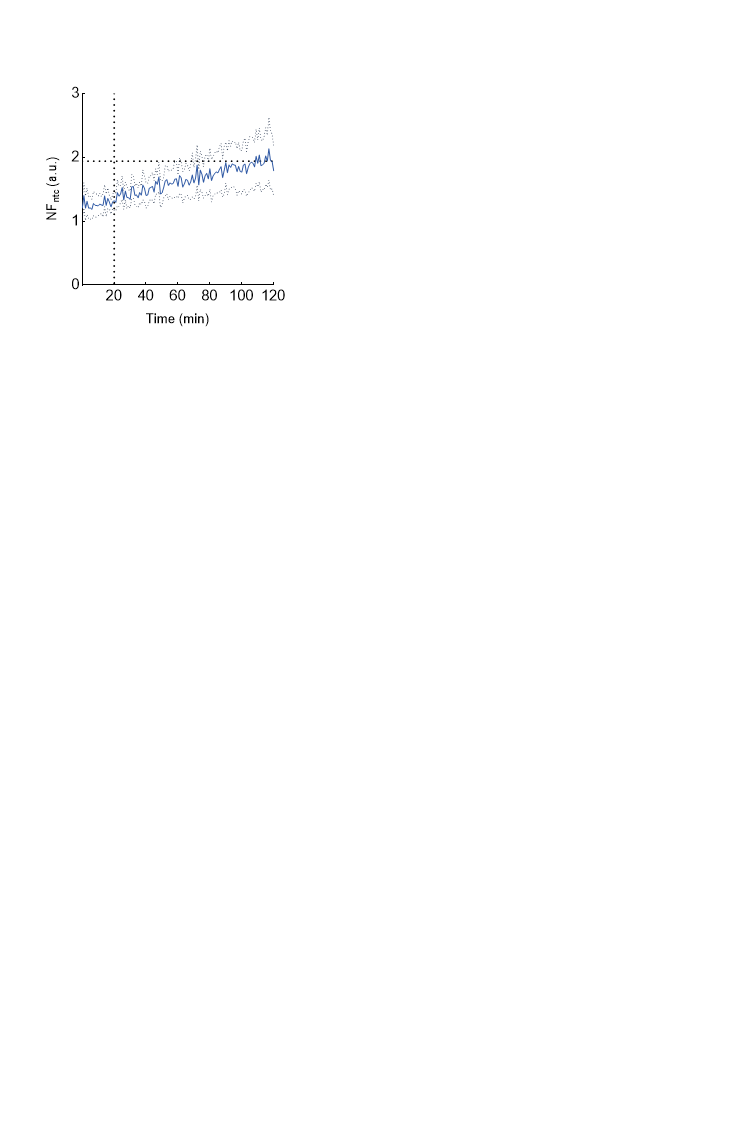


**S4 Fig. Determination of Limit of Blank (LoB) for the selected crRNA and assay conditions.** Normalized fluorescence values (NF_NTC_) over time (minutes) for ten negative samples were analyzed to determine the Limit of Blank (LoB). The X-axis dotted line represents the selected reading time for the assay. The Y-axis dotted line represents the calculated LoB = 1.92 a.u. Error bars represent the standard deviation of at least ten consecutive measurements.


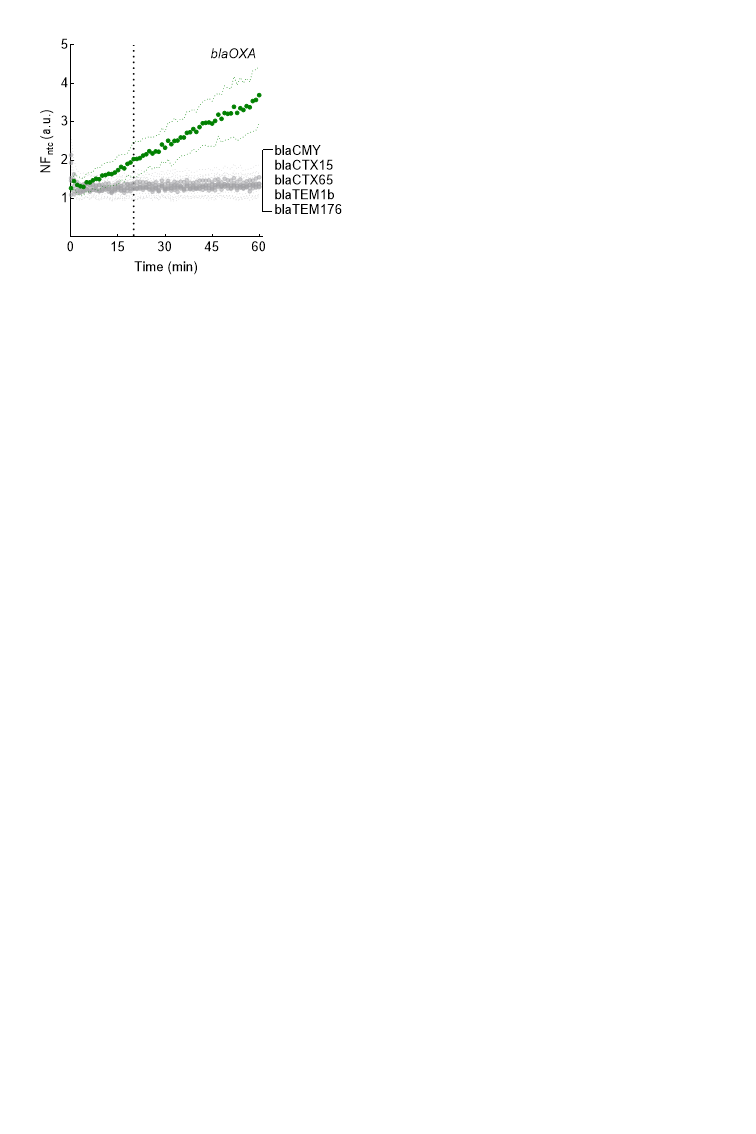


**S5 Fig. Assay specificity evaluation.** The optimized CRISPR-Cas-based assay was evaluated against different *E. coli* harboring other non-*bla_OXA-1_* genes. Across 60 minutes reading, only the *bla_OXA-1_* sample showed an increased normalized fluorescence. Dotted line in the X-axis represents a 20-minute cutoff. Each sample was evaluated in triplicate.
