# Supplementary material for: Thermal optimized PCR coupled to CRISPR-Cas12a for rapid detection of blaOXA-1 resistance gene
Source: PLoS One. 2026 May 15;21(5):e0337675. doi: 10.1371/journal.pone.0337675 (PMC13178884; doi:10.1371/journal.pone.0337675)
Supplement: Attachment — Submitted filename: Response_to_reviewers.docx [file pone.0337675.s005.docx]

**Reviewer #1: Thermal optimized PCR coupled to CRISPR-1 Cas12a for rapid detection of blaOXA-1 resistance gene**

**This study developed a CRISPR-Cas12a-based fluorescence method for the detection of blaOXA-1 gene, with a sensitivity of 8 CFU. Primers designed from consensus sequences of blaOXA-1 were employed in conventional PCR for initial optimization of conditions, followed by CRISPR-Cas detection.**

**Major comments**

The study does not convincingly prove the specificity of the assay. The authors should have tested their primers with diverse blaOXA-1-harbouring isolates, both E. coli and other Enterobacterales, and other ESBL- and MBL-encoding genes to prove the specificity of the method developed.

We agree that evaluating the assay against a broader panel of isolates would strengthen the study. In line with this statement, we stated in the manuscript that the principal limitation of this proof-of-concept study is the lack of diverse beta-lactamases-harboring strains.

However, as detailed in our discussion, the reported assay relies on a two-level specificity mechanism. (1) Primer annealing. Here our bioinformatic analysis of 42 other *blaOXA* groups revealed that the used primers either failed to align or showed low-coverage annealing, which will avoid unspecific beta-lactamases amplification; and (2) CRISPR-Cas12a discrimination. Even if non-specific amplification occurred, Cas12a activity depends on the presence of PAM sequencing and matching seed region to allow Cas12a activity. Our analysis confirmed that none of the other evaluated groups possessed this required combination.

To further address the concern regarding specificity against common beta-lactamases, we have performed a new experiment (now included as SX Fig). We evaluated the specificity of our primers and crRNA against a panel of available isolates harboring *blaCMY, blaCTX* and *blaTEM*. No amplification or CRISPR-mediated signal was observed for these non-target genes, confirming the discrimination capability of the reported assay.

What is the specific advantage of this method, when PCR methods for blaOXA variants are already available?

While different PCR-based methods for *blaOXA* detection have been reported, they often face significant implementation obstacles. Therefore, our assay offers two specific advantages:

1. Conventional PCR requires gel electrophoresis for visualization, which adds time and requires additional equipment. Conversely, real-time PCR needs high-cost, specialized equipment that is often unavailable in low-resource settings. By using CRISPR-Cas technology, our assay provides a rapid fluorescent readout compatible with basic equipment such as blue-light transilluminators or low-cost 3D-printed fluorometers, facilitating decentralized testing.
2. While isothermal techniques like LAMP or RPA are faster than standard PCR, they frequently suffer from non-specific amplification, complicated primer design, or limited reagent availability- Our assay depends on the well established and wide availability of PCR reagents while overcoming the speed bottleneck of the technique. Here, we demonstrated that PCR can be optimized to achieve amplification times competitive with isothermal assays. Our results showed a complete workflow from amplification to detection in 50 minutes.

A short paragraph summarizing this response has been added properly in the discussion section.

Further, the authors need to clearly define the goal (s) and the application of their work in the abstract and in the introduction section. It is only evident after section 2.4.3. (Spike-in pilot test) that the method was indeed designed to detect the target organisms (E. coli carrying blaOXA-1 gene) in chicken feces.

Thank you for your comment. We have updated the manuscript to clearly indicate the study goal.

In L217-223: It is stated here that the mismatches in primer binding sites might potentially affect detection of the variants of blaOXA gene. This being the case, is this method specific to only blaOXA-1 gene? Does this not limit the application of this method, since blaOXA genes are very diverse?

You are right that the high diversity of antimicrobial resistance genes presents a significant challenge for any molecular detection assay. In this context, regarding the implications for the mismatches and the application of the reported approach:

1. Our assay was designed to target the *blaOXA-1* family, which is one of the most prevalent among OXA-type enzymes. This family includes the *blaOXA-1* gene which is the most prevalent variant within this group. As we indicated in the results section, the *blaOXA-1* family showed a high level of conservation across its full-length sequence. This high conservation ensures that the assay is not limited to single genes but can detect most of the circulating variants of this family. It is important to highlight that focusing on relevant marker groups is a standard strategy for molecular diagnostics for rapid monitoring, providing first-line screening that can later be confirmed with high-resolution tests like NGS.
2. Regarding the mismatches observed for four variants (e.g. *blaOXA-47, blaOXA-392, blaOXA675, blaOXA1370*), a single mismatch was identified within the seed region. While previous studies suggest that single mismatches in the seed region may affect Cas12a-mediated cleavage, the presence of canonical PAM and perfect alignment across the rest of the crRNA sequence suggests that activity may be reduced but not necessarily abolished. Therefore, we have mentioned that experimental validation is required to determine the exact effect.

L165: Here, amplification product of 564 bp has been mentioned. What does this product refer to?

This PCR product is amplified using another forward primer. As indicated in the results section (S2 Fig). This PCR product contains the target region for the crRNA designed and evaluated in the study. We have added a sentence describing how this PCR product was obtained. This longer PCR product was used to evaluate if longer amplicons can be obtained with the modified PCR protocol.

5 x 10-3 attomoles of pure DNA: Please compare and discuss the sensitivity with pure DNA and the spiked bacterial cells (8 CFU/reaction). Alternatively, this may be represented as gene copies.

Thank you for your suggestion. Providing sensitivity as gene copy number allows for a more standardized comparison across different approaches. For our results, a sensitivity of 5 x 10-3 attomoles/reaction in reaction is equivalent to 3000 copies/reaction approximately, for 564-bp linear dsDNA. However, gene copy number calculation from cells can be complex. In our case the exact number of gene copies per bacteria is not determined and could vary if the gene is located in plasmids.

But the fact that our assay can detect as low as 8 CFU/reaction demonstrated that proposed approach is highly efficient and competitive with other CRISPR-Cas-based studies, as indicated in the discussion.

While purified DNA is useful to determine absolute molecular sensitivity of detection assays, we maintain that cell count (CFU) is a more relevant metric for clinical and environmental monitoring, since CFU number represents the target is real world samples.

We have added a statement in the results section to include the calculated gene copy number for comparison, and in the discussion about the relevance of these metrics.

Minor comments

Please label A & B in Figure 2

Thank you for your observation. Labels have been added.

Figure 3. Please move (A) to the next line

Thank you for your observation. Legend have been updated.

Reviewer #2: The study is simple yet performed with relevant outcomes. However, authors can address the following comments

1. What is the inclusion/exclusion criteria used while selecting 46 blaOXA-1 gene sequences?

We retrieved all available sequences reported as *bla_OXA-1_* family deposited in the indicated databases. From the NDARO, *bla_OXA-1_* family’s sequences were retrieved using the browse for the pathogen detection reference gene catalog.

2. Authors need to specify the version of tools used and parameter settings wherever applicable throughout the methodology

Thank you for your comment. It has been updated.

3. Times reported for different TRR with lab-made Taq (e.g., 0.6 °C/s = 83 minutes) differ from those with DreamTaq. Can authors clarify why total run times differ, given similar cycle numbers and temperatures?

While cycle numbers and temperatures were consistent for both enzymes, the total run times differed due to the holding time required per enzyme. For the commercial enzyme, holding times of 1s were enough to obtain target amplification. In contrast, for the lab-made Taq, 10 s is required to ensure target amplification. We hypothesized that this is due to the lower polymerization rate and lack of reaction buffer optimization. This is already mentioned in the discussion section.

4. Can authors provide alternative gel image for Figure 2A since the bands appear at different size range (could be due to imaging error)

We would like to clarify that the observed variations in band migration are not due to imaging errors, but rather a phenomenon associated with our staining protocol. For these assays, we utilized a pre-staining approach where SYBR Gold was mixed directly with the tracking buffer. It is documented in literature (<https://pubmed.ncbi.nlm.nih.gov/4354250/>) that intercalating dyes can affect DNA migration, particularly when the DNA concentration is low. This results in apparent shifts in migration even for PCR products of identical size. This effect is most pronounced in Fig 2A due to the variation in PCR product yield. In contrast, Fig 2B shows highly consistent band sizes. We have clarified how we stain the PCR product for visualization in gel electrophoresis, in the manuscript.

5. Two different plate readers were used. Were the instruments cross-calibrated to ensure comparable fluorescence readings?

Yes, we performed cross-measurements using identical sets of positive and non-template control on both instruments. We utilized a normalized fluorescent ratio to strategy minimizes the impact of different instrument technical configurations. We have added a SX Fig, which shows that there is not difference for signal output for the mentioned controls.

6. How was 10 blaOXA-1 negative E. coli isolates confirmed? Whether by PCR or WGS?

Thank you for your observation. All *E. coli* isolates used in our study were previously characterized by NGS. We have added a sentence indicating this.

7. Authors used a 564 bp purified amplification product as target for Target concentration curve. Why is this length different from the 282 bp region described earlier?

This 564-bp fragment contains the 282-bp target region used in our standardization experiments. For the target concentration curve, we preferred to avoid using the same length-amplicon as target to reduce the risk of carry-over contamination.

8. For amplification with lab-made enzyme cycle time was 10 s per step, while 1 s protocol was followed for DreamTaq. Can authors justify the difference?

We have provided justification for this difference in results and discussion sections. We hypothesized that the observed difference could be due to reaction buffer composition and enzyme processivity. Therefore, we suggested the target amplification using a lab-made Taq needs further conditions optimization.

Despite the difference in amplification times, when comparing standard and modified thermal protocols using only the lab-made enzyme (Fig 3C – mid and right lanes), we observed minimal differences in the detection limit. This suggests that the performance bottleneck is a result of the enzyme quality and reaction conditions rather than the modified thermal cycling protocol itself.

9. During spike test, OD600 was adjusted to 0.05 before serial dilutions. Was CFU conversion calibrated for this isolate or assumed from literature?

Reported CFU numbers were determined by counting colonies on the incubated LB plates prepared from the isolate used in the spike-in assay. The OD600 value of 0.05 before serial dilution was used in accordance with our standard laboratory protocol. Each dilution was plated, and after incubation, the resulting CFU counts were recorded. We have updated the methodology describing the spike-in assay to clarifying this point.

10. “From each selected dilution, 100 μL were transferred to 900 μL of the fecal suspension, then, 250 μL of each suspension was used for total DNA extraction, corresponding to an estimated 8 and 60 total CFUs, respectively.” Was extraction efficiency (recovery of DNA per CFU) evaluated or assumed?

We did not evaluated recovery of DNA per CFU. The values indicated correspond to a theoretical input of cells on the counted CFU/mL of the selected dilutions and the volume used for DNA extraction. We have added a statement to clarify this point.

11. “Extraction was performed using the ZymoBIOMICS™ DNA Miniprep Kit (Cat. N° D4300, Zymo Research), following the manufacturer's protocol.” Could authors compare kit-based extraction to simpler or cheaper methods that may be relevant for field deployment?

Thank you for your suggestion. We recognize that evaluating alternatives protocols for DNA extraction are important, especially to enable lab-to-field transition. As mentioned in the manuscript, the study goal was to provide a proof-of-concept of a molecular detection assay, by optimizing PCR thermal cycling to achieve speeds competitive with isothermal assays while maintaining the reliability of conventional PCR. We used a standardized, kit-based extraction to ensure high-quality DNA input.

12. For spiked samples, authors used the modified PCR with DreamTaq and subsequent CRISPR detection. Did authors also test lab-produced Taq in the fecal matrix, and if not, why?

Thank you for your observation. Due to differences in analytical sensitivity between commercial and the lab-made Taq (Fig 3C), we chose only the commercial enzyme to performed the spike-in assay. Considering that complex biological matrices, such as the poultry fecal suspension used in our spike-in assays, could have various PCR inhibitors that can further reduce amplification efficiency
