# Supplementary material for: Thermal optimized PCR coupled to CRISPR-Cas12a for rapid detection of blaOXA-1 resistance gene
Source: PLoS One. 2026 May 15;21(5):e0337675. doi: 10.1371/journal.pone.0337675 (PMC13178884; doi:10.1371/journal.pone.0337675)
Supplement: Attachment — Submitted filename: Response to reviewers.docx [file pone.0337675.s006.docx]

**Response to reviewers**

Reviewer #1: The authors have responded to the queries raised in the previous review. Yet, the application of blaOXA-1 group -specific detection method has limited applicability in view of the fact that this does not cover the most common blaOXA gene(s) either in E. coli or other Gram-negative pathogens such as Acinetobacter baumannii.

Thank you for this perspective. We agree that blaOXA genes are remarkably diverse and that different variants are more prevalent depending of the host species, geographical area and clinical/environmental context. However, we maintain that blaOXA-1 represents a priority target for the following reasons:

- High prevalence in other clinically relevant Gram-negative: Recent literature confirms high frequencies exceeding 50% in clinical *Klebsiella pneumoniae* isolates (Meng *et al.,* 2023), and identifying blaOXA-1-carrying plasmids as the most abundant (57.93%) in *E. coli* isolates (Wu *et al.,* 2024). In both studies, independently of the geographic area.
- Co-presence with other ARG markers: blaOXA-1 is frequently associated with class 1 integrons and co-occurs with other relevant resistance markers such as blaXTX-M-15 and aac(6’)-Ib-cr.
- Presence in environmental and food samples: Some studies have reported high prevalence of blaOXA-1 in soil and manure samples, 45.16% (Laconi *et al.,* 2021), and in *E.coli* isolated from poultry meat, 40.54% (Ali 2025).

Finally, we acknowledge that this assay does not cover all relevant and high frequent OXA-carbapanemases. However, as a proof-of-concept, we intended to develop a rapid, user-friendly and affordable tool for monitoring this emerging resistance gene.

These statements and references have been added to the manuscript.

The application of an in-house polymerase is something unique in this study, although it is not clear how the results obtained using an in-house polymerase can be reproduced in other laboratories beyond the geographic region where this study was performed. Further, the authors have stated in the manuscript that this polymerase had a lower efficiency (processivity) compared to commercial polymerase (s). In my opinion, the section describing in-house polymerase may be removed, as the data/results are relevant only to the laboratory employing it.

Thank you for your perspective. We maintain that including the local-produced DNA polymerase results is important for contributing to the democratization of molecular diagnostics. By providing full standardized protocols (Reference 30) and evidence of their implementation, we demonstrate that molecular detection can be achieved based on local efforts. Although the Taq DNA polymerase showed a lower performance compared to a commercial alternative, its inclusion serves as a proof-of-concept for local enzyme production. We stated that further optimization is needed and does not discard the utility of local-produced enzymes for implementing molecular detection assays, even in emergency contexts such as future pandemics as we stated in lines 468-470.

A few suggestions are below for authors’ consideration

75: Please correct as ..”needing long amplification times.

Thank you. It has been edited.

L96-97: Please rephrase this sentence.

Thank you. It has been rephrased.

Please write blaOXA correctly throughout the manuscript. OXA should be subscript of bla and in non-italics.

Thank you. It has been modified.

L133: The dNTP concentration of 0.5 nM seems to be too low. Please check. Similarly, the Taq concentration is generally stated in units, but you have indicated in ng/µL.

Thank you. Yes, it was a typo. The correct unit is mM. Regarding the Taq Polymerase locally produced, the units have been not determined. Therefore, we express the working enzyme concentration as ng/µL.

L163-164: Please write bla genes in correct format.

Thank you. It has been corrected.

L208: Please provide the strain number/designation of blaOXA-1 isolate you used in this study

Thank you. It has been added.

L208: Replace “cultivated” with “grown”

Thank you. It has been replaced.

L211: Seeded? It appears that you performed a spread plate. However, spread plate method employs 100 microliters, not 50.

Yes, as it is indicated in the manuscript, we used 50 μL for the assay. CFU counts were calculated using the corresponding volume and dilutions factors.

L214-216; Please rephrase the sentence.

Thank you. It has been rephrased.

L446-451: Agreed that qPCR and NGS are expensive. In what way your method is less expensive or technically less demanding than conventional PCR?. This method also requires PCR amplification, followed by fluorescence detection.

Thank you for the question. While our method utilizes PCR amplification and fluorescence detection, it remains significantly more straightforward than conventional PCR. The main advantage is the elimination of gel electrophoresis. By removing the need to visualize and confirm targets on a gel, we drastically reduce turnaround time and remove the requirement for additional equipment like electrophoresis chambers, and power supplies. This statement has been added to the manuscript.

Reviewer #2: (No Response)
